# Melatonin Prevents T Lymphocyte Infiltration to the Kidneys of Hypertensive Rats, Induced by a High-Salt Diet, by Preventing the Expression of CXCR3 Ligand Chemokines

**DOI:** 10.3390/nu13103577

**Published:** 2021-10-13

**Authors:** Ariel Bier, Rawan Khashab, Yehonatan Sharabi, Ehud Grossman, Avshalom Leibowitz

**Affiliations:** 1 Medicine D, The Chaim Sheba Medical Center, Tel-Hashomer 5262000, Israel; arielbier@gmail.com (A.B.); Yehonatan.Sharabi@sheba.health.gov.il (Y.S.); Ehud.Grossman@sheba.health.gov.il (E.G.); 2 Hypertension Unit, the Chaim Sheba Medical Center, Tel-Hashomer 5262000, Israel; rawankhasbab@gmail.com; 3 Sackler Faculty of Medicine, Tel-Aviv University, Tel-Aviv 6997801, Israel

**Keywords:** melatonin, salt, T-lymphocytes, hypertension, kidney, CXCL 9, CXCL 10, CXCL 11

## Abstract

In a previous study, we demonstrated that melatonin prevents kidney damage in a salt-induced hypertension model by decreasing oxidative stress. We hypothesized that this effect involves melatonin’s immunomodulatory properties. In vivo Study-Dahl salt-sensitive (DSS) rats were fed normal chow, a high-salt diet (HSD), or a HSD and melatonin (30 mg/kg/day) in their water for eight weeks. Kidneys were harvested for immediate lymphocyte isolation and characterization by Flow cytometry (CD3+CD4+ and CD3+CD8+) and for lymphocyte chemoattractant (mainly CXCL chemokines) gene expression studies. In vitro study-rat mesangial cells (RMC) were cultured in a high-salt medium without and with melatonin. A HSD was associated with significant renal infiltration of CD4+ and CD8+ T lymphocytes compared to control. Melatonin significantly reduced renal lymphocyte infiltration. A HSD significantly increased mRNA expression of CXCL chemokines. Adding melatonin to the HSD abolished this effect. Treating RMC cells with salt increased the expression of CXCL10 and CXCL11 but not CXCL9. Adding melatonin to the culture media prevented this increase. Treating HSD-fed rats with melatonin decreased renal lymphocyte chemoattractant mRNA expression and is associated with significantly reducing renal T lymphocyte infiltration. Salt may have a direct effect on chemokine-producing renal cells, which is blunted by melatonin treatment.

## 1. Introduction

Hypertension (HTN) is a leading modifiable cardiovascular risk factor. Due to population aging, the prevalence of HTN is steadily increasing, reaching up to 30% in advanced ages [1]. Despite various medical therapeutic options, worldwide control of HTN is poor. The consequences of uncontrolled HTN are devastating, creating a huge negative impact on public health [2].

Various behavioral, environmental and genetic factors are involved in the pathogenesis of HTN. The high salt content in Western diets has been implicated as a major contributor for HTN and is associated with increased morbidity and mortality. Guidelines and public initiatives recommend reducing salt intake for the general population. However, not every individual may benefit this restriction, as the pathophysiology connecting salt sensitivity to HTN is not fully understood and needs improved mechanistic understanding [3]. Thus, new strategies are crucial for better HTN understanding and control, making new research paradigms an immediate need.

Inflammation and oxidative stress have been found to be involved in the pathogenesis of essential HTN. While researching for the triggers initiating and continuing these processes, many experimental HTN models suggest the involvement of the adaptive immune response [4].

Clues connecting adaptive immunity to HTN have already existed since the 1960s. However, only lately, with a better understanding of the immune system, progress could be achieved in this direction. A pioneering study by Guzik et al. demonstrated that angiotensin (Ang) II or deoxycorticosterone (DOCA) salt HTN was blunted in rag1−/− mice, which are deficient in T and B lymphocytes, and adoptive transfer of T, but not B cells, could restore the BP response [5]. Since this paper, various adoptive immunity components were found to be relevant to the pathogenesis of HTN in most of the common animal models. The role of T cells in the salt-induced HTN model has also been extensively studied. In vitro studies show that salt may push naïve T cell differentiation into TH17 cells [6]. Numerous in vivo works in high-salt diet- (HSD) induced HTN models, such as in Dhal salt-sensitive (DSS) rats, show extensive renal T cells infiltration [7,8,9].

Among the many physiological processes, melatonin’s involvement with blood pressure regulation is somewhat intuitive. The circadian pattern of blood pressure encouraged extensive research regarding melatonin and HTN. Many works in the past, in humans and in experimental models, connected low melatonin levels to HTN [10]. There are some clinical data suggesting that melatonin supplementation may treat HTN, mainly for patients with nocturnal HTN [11]. The inconsistency of these data, however, raises questions on whether non-circadian melatonin properties are also involved in blood pressure regulation.

The anti-inflammatory and anti-oxidative properties of melatonin have suggested that melatonin interacts with the immune system. Emerging data have implicated the role of melatonin signaling in T cell development, activation, differentiation and memory. There is evidence that melatonin membrane and nuclear receptors are present on T cells [12,13]. Melatonin affects the production of many cytokines involved in immune response, such as interferon (IFN)—γ which is a pivot regulator of Th1 response [14].

Oxidative stress and T cell response, according to some researchers, work in concert promoting the development of HTN and organ hypertensive damage [15]. In our previous work, we demonstrated that melatonin decreases local (renal) oxidative stress and prevents salt-induced renal fibrosis and proteinuria in a salt-sensitive HTN rat model (the DSS model) [16]. Since oxidative stress has a pivotal role in HTN-associated adaptive immune response, we hypothesized that melatonin’s immunomodulatory properties are responsible for its protective role in HSD-induced HTN.

The aim of this study is to evaluate whether melatonin decreases the salt-induced T cell response and to elucidate the mechanisms responsible for this effect.

## 2. Materials and Methods

### 2.1. Animals and Study Protocol

The study protocol was approved by the institutional animal ethics committee in Sheba medical center at Israel. Their state of health was monitored by measuring body weight and a daily general observation for movement and health condition for each animal. Male Dahl salt-sensitive rats, four weeks old (RGD Cat# 1582190, RRID: RGD_1582190), were housed in in an animal facility in regular cages (two rats per cage) at 22 °C with a 14 h light/10 h dark cycle. The rats were maintained on a normal salt diet and were given tap water to drink ad libitum for an acclimation period of five days. The rats were then divided into three groups (*n* = 8), according to diet, over an 8 week period. According to the ethics committee, animals which showed signs of illness and suffering were to be sacrificed.

The control group was fed a standard rat chow diet (2018 SC; Teklad Envigo, Madison, WI, USA) and tap water; the high-salt diet (HSD) group was fed a matched enriched salt diet (4%) (TD.120485; Teklad Envigo, Madison, WI, USA) and tap water, and the melatonin group was fed the same enriched salt diet (4%) with melatonin (M5250; Sigma, Rehovot, Israel) (30 mg/kg/day) in their drinking water. Body weight and water consumption was measured periodically. The correct melatonin amount was dissolved in the match water needs per each cage in order to deliver the correct dose for each animal. Day zero is defined as the day of starting the HSD and melatonin consumption. At the end of the study, the rats were sacrificed by deep anesthetization with 3% isoflurane (depth of anesthesia confirmed by rear foot squeezing) and the kidneys were harvested. Both kidneys were removed and hemisected. The kidneys were taken for flow cytometry analysis except for several portions which were quickly removed and snap-frozen in liquid N2 and stored at −80 °C. Frozen tissues were used for RNA extraction.

### 2.2. Real-Time Quantitative Reverse Transcription PCR

mRNA expression levels of genes were ascertained in the kidney tissue by real-time quantitative reverse transcriptase-PCR (qRT-PCR). Total RNA was extracted from the kidney tissue using a NucleoSpin RNA Kit (MACHERY-NAGEL, Düren, Germany). Reverse transcription was performed using an Applied Biosystems High-Capacity cDNA Reverse Transcription Kit (Applied Biosystems, Foster City, CA, USA). qRT-PCR reactions were performed using the Power SYBR Green PCR Master Mix (Applied Biosystems, Warrington, UK) and the Applied Biosystems 7500 real-time PCR system. The cycling conditions were: first single step of 95 °C for 0.20 min and then 40 cycles of melt stage for 3 s at 95 °C and an extended stage of 30 s at 60 °C. At the end of the 40 cycles, a melt curve stage was performed. The ribosomal protein lateral stalk subunit P0 (Rplp0) mRNA was used as an internal control. The primers are listed in Table 1.

### 2.3. T Cells Isolation from Kidneys

After removing the renal capsule, the two kidneys were minced and teased with the plunger of a 2 mL syringe through a 100 μm cell strainer in RPMI 1640 containing 2 mM L-glutamine, 10% FBS, 10 ug/mL DNase 1 (Stem Cells technologies, Cambridge, MA, USA) and 0.1% collagenase type IV (CLS 4, Worthington Decatur, AL, USA). The solution was incubated for 25 min at 37 °C. Washing solution (DPBS/2% FBS/2 mM EDTA) was then added to the kidney homogenate to reach 45 mL and was filtered through a 70 μm cell strainer. The solution was centrifuged at 400× *g*/7 min. and the pellet was suspended in 5 mL washing solution and filtered through a 40 μm cell strainer and recentrifuged at 400× *g*/7 min. The pellet was resuspended in 3 mL FBS (10 mM EDTA) and layered over Histopaque-1083 (Sigma, Rehovot, Israel), and centrifuged at 20 °C at 400× *g*/30 min with the brake off. The mononuclear cell layer resting above the Histopaque was removed, washed with washing solution twice and suspended in 5 mL washing solution for flow cytometric analysis.

### 2.4. Flow Cytometry

From each group, kidneys from 5–7 rats were analyzed. For each sample, 1 × 10^6^ cells were taken. Isolated mononuclear cells were incubated with extracellular markers: anti-CD3 (Thermo Fisher Scientific Cat# 12-0030-82, RRID:AB_465493) and its isotype control (Thermo Fisher Scientific Cat# 12-4742-41, RRID:AB_10753770), anti-CD4 (Thermo Fisher Scientific Cat# 17-0040-80, RRID:AB_1210581) and its isotype control (Thermo Fisher Scientific Cat# 17-4724-81, RRID:AB_470188), anti-CD8 (Thermo Fisher Scientific Cat# 25-0084-82, RRID:AB_10548361) and its isotype control (Thermo Fisher Scientific Cat# 25-4714-80, RRID:AB_657914). All cells were analyzed by flow cytometry (BD FACSCalibur Flow Cytometry System, RRID:SCR_000401) using the FlowJo software (FlowJo, RRID:SCR_008520).

### 2.5. Cell Culture

The mesangial rat cell line, RMC (ATCC Cat# CRL-2573, RRID: CVCL_0506), was obtained from ATCC^®^. Cell were cultured in Dulbecco’s modified Eagle’s medium (01-055-1A, biological industries, Beit HaEmek, Israel) with 4 mM L-glutamine adjusted to contain 1.5 g/L sodium bicarbonate and 4.5 g/L glucose and supplemented with 0.4 mg/mL G418 and 15% fetal bovine serum. Melatonin was obtained from Sigma (M5250 Rehovot, Israel). A 250 mM melatonin stock was made in ethanol. Cells were cultured in normal or high-salt (adding 80 mM NaCl to the medium) conditions without or with melatonin (0.5 mM). Melatonin treatment was timed 45 min before adding NaCl to the medium. All experiments were in 6 well plates with 3 × 10^5^ cells per well, and were performed by 6 independent trials.

### 2.6. Statistical Analysis

Data are presented as mean ± S.E.M. Statistical analyses were performed using IBM SPSS Statistics, RRID: SCR_019096. One-way analysis of variance (ANOVA) and the post-hoc Tukey method examined the differences between groups. Real-time qPCR data were analyzed using DataAssist, RRID: SCR_014969.

## 3. Results

### 3.1. Melatonin Protects Rats Consuming a HSD

**Figure 1:**
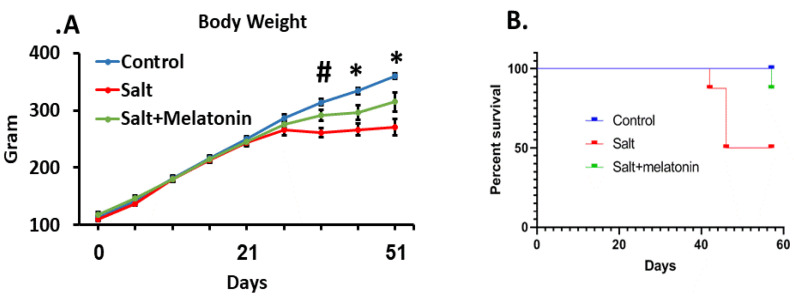
Melatonin ameliorated blood pressure and survival in HSD. DSS Rats were treated for 9 weeks with HSD with and without melatonin. High-salt diet reduced body weight from day 36 and melatonin moderated this effect (**A**). HSD increased mortality rate from day 40. Treating HSD-fed rats with melatonin improve their survival. (**B**). (*n* = 8) *-*p* ≤ 0.05 salt and salt+melatonin versus control. #-*p* ≤ 0.05 salt versus control and salt+melatonin. HSD—high-salt diet.

The rats’ age during this study was 4–13 weeks. At this age, the rats’ wellbeing is indicated by weight gain which was exhibited by the control group [17]. However, the HSD group reached a plateau at day 30 and the addition of melatonin to the HSD ameliorated this phenomenon (Figure 1A). The HSD group exhibited a high mortality rate, where 50% of the rats died or had a clinical condition that required euthanasia according to the animal ethics committee rules, during the experimental period. Melatonin supplements prevented the rats’ mortality significantly, where only one out of eight rats died at day 58, at the end of the experimental period (Figure 1B).

### 3.2. Melatonin Prevents Kidney T Cell Infiltration Induced by HSD

**Figure 2:**
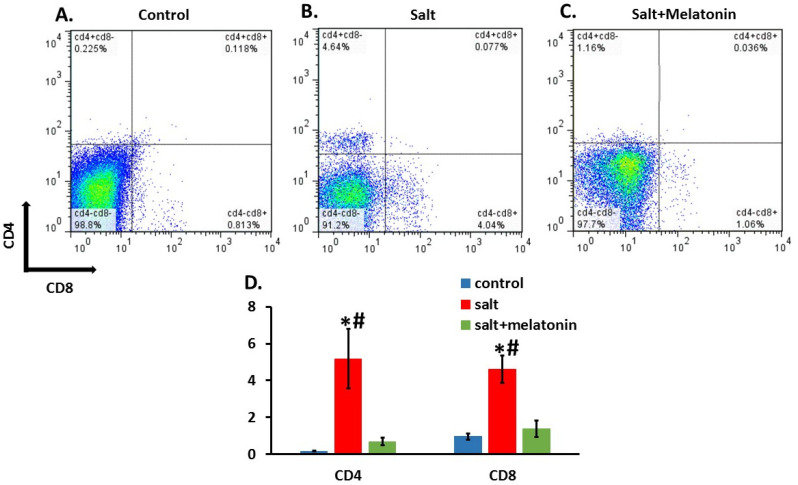
Melatonin prevent T cells infiltration to the kidney. T cells were isolated from rats’ kidneys and were gated for CD3. The CD3-positive cells were gated for CD4 and CD8. Melatonin (**C**) reduced both CD3+CD4+ and CD3+CD8+ as compart to HSD without melatonin (**B**) with no different from the control group (**A**). An analysis for all rats is shown in (**D**) (*n* = 7–8). HSD—high-salt diet. *-*p* ≤ 0.05 versus control. #-*p* ≤ 0.05 versus salt+melatonin.

We isolated cells from the kidney and gated them to the T cell marker, CD3. CD3+ cells were gated to CD4 and CD8. Both CD3+CD4+ and CD3+CD8+ were significantly higher in the HSD (5.18 ± 1.62 and 4.6 ± 0.75%, respectively) compared to the control (0.16 ± 0.02 and 0.95 ± 0.15%, respectively) and melatonin supplementation to the HSD reduced both CD3+CD4+ and CD3+CD8+ cells back to control levels (0.68 ± 0.21 and 1.37 ± 0.45, respectively) (Figure 2A–D).

### 3.3. Melatonin Reduces Certain Kidney Chemokines Which Were Upregulated in HSD

The recruitment of T cells to inflammatory tissues is regulated by local chemokine expression. We therefore measured the expression of two chemokine families in the rats’ kidneys: the chemokine (C-X-C motif) ligand (CXCL) family and the chemokine (C-C motif) ligand (CCL) family.

**Figure 3:**
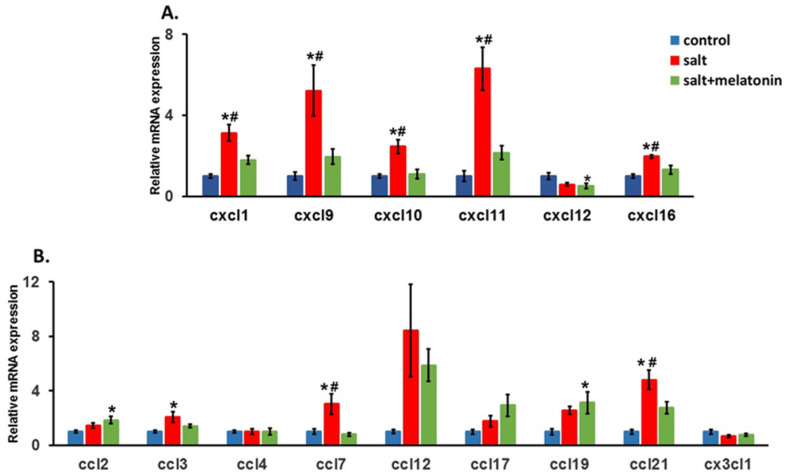
Upregulation of kidney’s chemokines in HSD with and without melatonin. The expression of chemokine (C-X-C motif) ligand (CXCL) family (**A**), chemokine (C-C motif) ligand family and CX3CL1 (**B**) were determined in the rats. (*n* = 7–8) *-*p* ≤ 0.05 versus control. #-*p* ≤ 0.05 versus salt+melatonin. HSD—high-salt diet.

For the CXCL family, CXCL 9, 10 and 11 which attract T cells to their target tissue using the CXCR3 receptor on the T cells, were significantly upregulated in HSD rats. The supplementation of melatonin to the HSD reduced their expression back to control levels. The same pattern was detected for CXCL 1 and 16. However for CXCL12, no upregulation was detected in the HSD (Figure 3A).

For the CCL family, CCL 2, 4, 12, 17, 19 and CX3CL1 showed no upregulation in HSD. CCL3 was upregulated in HSD, but no significant change was detected with the addition of melatonin to the diet. For CCL7, a significant upregulation in the HSD and a significant downregulation with the addition of melatonin to the HSD was detected (Figure 3B).

### 3.4. The Direct Effect of Melatonin on the RMC Mesangial Cell Line

We then focused on CXCL9, 10 and 11 which attract T cells by binding to the receptor CXCR3. We determined the expression of these chemokines in RMC cells which are a rat mesangial cell line.

**Figure 4:**
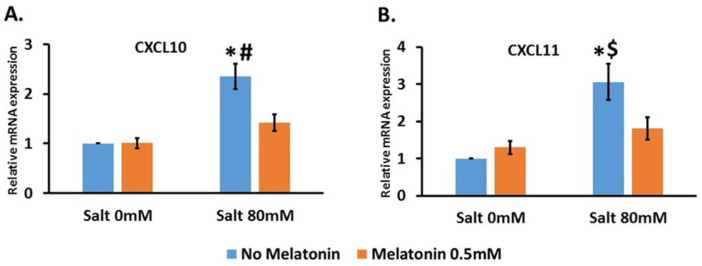
Salt upregulated CXCL10 and CXCL11 expression in mesangial cell line where melatonin treatment downregulated its expression. RMC (rats’ mesangial cell line) were treated with salt 80 mM with and without 0.5 mM melatonin. CXCL10 (**A**) and CXCL11 (**B**) were upregulated by salt treatment and the addition of melatonin reduce this upregulation. The graphs present 6 independent trials. *-*p* ≤ 0.001 versus control #-*p* ≤ 0.005 versus salt 80 mM and melatonin 0.5 mM. $-*p* ≤ 0.05 versus salt 80 mM and melatonin 0.5 mM.

We treated the cells with 80 mM NaCl without and with 0.5 mM melatonin. This concentration of NaCl significantly increased the expression of CXCR10 by 2.4 ± 0.26-fold compared to the control, and the addition of 0.5 mM melatonin significantly decreased this expression to 1.4 ± 0.17-fold compared to the control (Figure 4A). For CXCL11, 80 mM NaCl significantly increased the expression by 3 ± 0.48-fold compared to the control, and the addition of 0.5 mM melatonin significantly decreased this expression to 1.8 ± 0.3-fold compared to the control (Figure 4B). CXCL9 was not detected in RMC cells.

## 4. Discussion

The beneficial effect of melatonin on HTN was demonstrated in previous studies and was suggested as a possible course of action for treatment-resistant HTN [18]. In our first study, we focused on oxidative stress, and revealed that melatonin reduces oxidative stress induced by HSD. In the present study, we focused on the adaptive immune effect and showed that melatonin improves the survival of HSD-fed rats. This effect is associated with a reduction in T cells infiltrating the kidney and the downregulation of kidney chemokine expression.

Historical data demonstrate that DSS rats consuming a HSD develop severe HTN and exhibit higher mortality rates [19]. Dahl himself reported while establishing the model that when DSS rats were placed on a HSD (8% NaCl) at weaning (21–23 days of age), they rapidly developed HTN and all died by the 16th week of salt feeding [20]. Later on, after some modifications of the strain, Rapp et al. reported that all rats were dead within eight weeks of the HSD (8% NaCl) [21]. The rats in our study had a high rate of mortality yet did not reach 100%, probably due to the lower percentage (4%) of salt in their diet. Nevertheless, it is obvious that melatonin supplementation significantly prevented rat mortality.

Gaining weight is a marker of the rats’ wellbeing at the age of 4–13 weeks. DSS rats originate from Sprague Dawley, which is a well-characterized strain. At the age of rats that we used in our experiments, the rats are supposed to gain weight consistently [22]. In many animal studies, weight reduction is considered as one of the “end points” of the experiments [23]. From several studies it is clear that a HSD in DSS rats results in lower weight gain rates compared to controls [24,25]. In concordance with these studies, we also observed weight differences between the groups. Here, the rats did not gain weight after four weeks of HSD. Melatonin stopped this effect of the HSD as well, as the rats which were treated with melatonin continued to gain weight, albeit less than the controls.

The ability of melatonin to reduce blood pressure in animal models was discovered in the late seventies of the 20th century [26,27,28]. Since then, it was reported in several other models, such as that a high-fructose diet induces metabolic syndrome in spontaneously hypertensive rats [29,30]. Our studies in this field are the first to show that melatonin plays a role in salt-induced HTN by preventing organ damage and reducing mortality.

In this study, we have shown that a HSD induces T lymphocyte infiltration to the kidney, a process which has been described in DSS rats mainly by Mattson’s group [31,32,33]. The pivotal role of this infiltration process in the DSS model has been demonstrated by a recent study which observed that T-cell-deficient DSS rats are protected from HTN induced by a HSD and a splenocyte transfer exacerbates salt-sensitive HTN [34].

Melatonin can affect T cells in various aspects (for review, see [35]). This study has shown for the first time that melatonin reduces the attraction of both CD4+ and CD8+ T cells to the kidney in HSD. This effect of melatonin may be the mechanism underlining its beneficial effect in this model.

The ability of melatonin to reduce T cell infiltration has been described in the experimental autoimmune encephalomyelitis (EAE) mice model. In EAE, melatonin was shown to reduce the infiltration of CD4+ T cells and Th17 cells into the CNS [36,37].

T cell attraction to a specific tissue is mainly regulated by local high concentrations of chemokines which are chemotactic cytokines that control the migratory patterns and positioning of immune cells. Two large chemokine families that induce T cell infiltration are the C-X-C motif ligand (CXCL) and the C-C motif ligand (CCL) chemokine families [38].

In our study, we did not find a constant pattern in the CCL family’s response to a HSD and to melatonin. Out of the nine chemokines in this family, only CCL7 and CLL21 were observed to increase their expression due to HSD, and melatonin supplementation reduced it. A HSD increased CCL3′s expression, but no reduction was detected in the melatonin treated rats. A recent study in the same model has shown that a HSD upregulates CCL2 in the kidney by using the RNA-Seq method [39]. However, this study tested the levels on days 3 and 21 after the diet started. In our study, we tested the levels after eight weeks. It is possible that chemokine expression changes during the HSD period.

Conversely, the levels of most of the CXCL family, including CXCL 1, 9, 10, 11 and 16, increased in the HSD-treated rats and melatonin supplementation abolished this increment. Only CXCL12 showed no increase in response to HSD.

CXCL9, CXCL10 and CXCL11, named “interferon-inducible CXC chemokine receptor 3 ligands”, are induced by IFN-γ and are the ligands of the CXC chemokine receptor 3 (CXCR3). In vivo studies have demonstrated their importance in the activation and recruitment of T lymphocytes to the target organ [40,41,42]. Youn et al. showed, in human hypertensive patients, both renal infiltration of T cells and elevated circulating levels of all the three CXCR3 chemokines, suggesting that CXCR3 and its ligands are relevant for T cells involvement in human HTN [43]. Another study has demonstrated that CXCL10 is elevated in hypertensive patients, and its levels are correlated to their blood pressure values [44].

Due to these studies, we focused on CXCL9, CXCL10 and CXCL11. We cultured the mesangial rat cell line and treated them with 80 mM NaCl and 0.5 mM melatonin. We found that CXCL 10 and 11 are upregulated in the cells and melatonin abolishes this elevation. Our results indicate that the effect of salt in elevating CXCL 10 and 11, and the opposite effect of melatonin, were both directly detected in mesangial cells. In contrast, CXCL9 was not detected in these cells.

Our study has limitations. In our FACS results, many of the CD3+ were double negative for both CD4 and CD8. The same results were seen in Mattson’s study from 2010 [31]. However, in more recent studies from Mattson’s group, the CD3+CD4-CD8- cells were very few [32,33,45,46]. The significant decrease in the double negative CD3 cells could be a result of adding the CD45 marker and only analyzing the cells which were CD45 positive, which is what was carried out in the later studies of Mattson. It is possible that the large population of the CD3+CD4-CD8- is an artifact. However, no artifact is known for the CD3+CD4+ and the CD3+CD8+ cell population.

## 5. Conclusions

In conclusion, a high-salt diet is associated with T cell infiltration into the kidneys of DSS rats. Salt also increased the expression of specific T cell chemoattractants in the kidney. Treatment with melatonin (30 mg/kg/day) abolished these effects (melatonin reduces HSD-induced infiltration of T cells into the kidneys along with a reduction in CXCL3 ligand chemokine expression).

Our in vitro study demonstrates that salt and melatonin may regulate chemoattractant expression by directly affecting kidney cells.

## Figures and Tables

**Table 1:** List of rats primers used in this study.

Gene Name	Forward	Reverse
Cxcl1	TGCTAAAGGGTGTCCCCAAG	TTGTCAGAAGCCAGCGTTCA
Cxcl9	TGTGGAGTTCGAGGAACCCT	ACCCTTGCTGAATCTGGGTC
Cxcl10	CCGCATGTTGAGATCATTGCC	CTAGCCGCACACTGGGTAAA
Cxcl11	TGATCATCTGGGCCACAACG	TGAGCCTTCAGGGTAACAATCA
Cxcl12	CCCCTGCCGATTCTTTGAGA	CTTGAGCCTCTTGTTTAAGGCT
Cxcl16	TTATCAGGTTCCAGTTGCAGTCC	GGTACTGGCTTGAGGCACAT
Ccl2	TGTCTCAGCCAGATGCAGTT	CAGCCGACTCATTGGGATCA
Ccl3	GCTTCTCCTATGGACGGCAA	CTTGGTCAGGAAAATGACACCC
Ccl4	CAGCACCAATAGGCTCTGAC	CTGGGGTCGGCACAGATTT
Ccl7	CCCTGGGAAGCTGTTATCTTCA	CCCCTTAGGACCGTAGTCCA
Ccl12	CCGGGAAGCTGTGATCTTCA	CTATCGCACTGTCCATGGGG
Ccl17	CTGCTCGAGCCACCAATGTA	GACAGTCTCAAACACGATGGC
Ccl19	TTCCTCCAAGAGCAAAGGCG	ACTCACGTTCACACCGACTC
Ccl21	ACAGGAAGCAAGAACCGAGC	TCTGTCTGTTCAGTCCCCTTG
Cx3cl1	GCCATCATCCTGGAGACGAG	ATGGCGTCTTGGACCCATTT
Rplp0-B	GAACATCTCCCCCTTCTCCTTC	ATTGCGGACACCCTCTAGGAA

## Data Availability

The data presented in this study are available on request from the corresponding author.
